# Immune Stimulation *via* Wounding Alters Chemical Profiles of Adult *Tribolium castaneum*

**DOI:** 10.1007/s10886-022-01395-x

**Published:** 2022-12-21

**Authors:** Lai Ka Lo, Reshma R, Lisa Johanna Tewes, Barbara Milutinović, Caroline Müller, Joachim Kurtz

**Affiliations:** 1 Institute for Evolution and Biodiversity, University of Münster, Hüfferstr. 1, 48149 Münster, Germany; 2 Department of Chemical Ecology, Bielefeld University, Universitätsstr. 25, 33615 Bielefeld, Germany

**Keywords:** Group-living insect, Immune defense, Benzoquinones, Cuticular hydrocarbons (CHC), Immune status signaling

## Abstract

Group-living individuals experience immense risk of disease transmission and parasite infection. In social and in some non-social insects, disease control with immunomodulation arises not only *via* individual immune defenses, but also *via* infochemicals such as contact cues and (defensive) volatiles to mount a group-level immunity. However, little is known about whether activation of the immune system elicits changes in chemical phenotypes, which may mediate these responses. We here asked whether individual immune experience resulting from wounding or injection of heat-killed *Bacillus thuringiensis* (priming) leads to changes in the chemical profiles of female and male adult red flour beetles, *Tribolium castaneum*, which are non-social but gregarious. We analyzed insect extracts using GC-FID to study the chemical composition of (1) cuticular hydrocarbons (CHCs) as candidates for the transfer of immunity-related information between individuals *via* contact, and (2) stink gland secretions, with analysis of benzoquinones as main active compounds regulating ‘external immunity’. Despite a pronounced sexual dimorphism in CHC profiles, wounding stimulation led to similar profile changes in males and females with increases in the proportion of methyl-branched alkanes compared to naïve beetles. While changes in the overall secretion profiles were less pronounced, absolute amounts of benzoquinones were transiently elevated in wounded compared to naïve females. Responses to priming were insignificant in CHCs and secretions. We suggest that changes in different infochemicals after wounding may mediate immune status signaling in the context of both internal and external immune responses in groups of this non-social insect, thus showing parallels to social immunity.

**Supplementary Information:**

The online version contains supplementary material available at 10.1007/s10886-022-01395-x.

## Introduction

In group-living animals, increased contacts of individuals facilitate the spread of diseases (Kappeler et al. 2015). Thus, the individual ability to respond to immune stimuli and limit infection, but also the recognition of the health-status of group members are vital (Kappeler et al. 2015). Especially eusocial insect species show remarkable physiological and/or behavioral group-level responses to disease, such as distancing (Stockmaier et al. 2021) or hygiene behaviors (Pull et al. 2018; Rosengaus et al. 1998) leading to ‘social immunity’ (Cremer et al. 2018; Liu et al. 2019). Insight into individual and group-level immunity across insect groups with different types of social structure is highly valuable. However, there have been only a few studies of the phenomenon of social transfer of immunity in non-social insects (Gallagher et al. 2018), and its underlying mechanisms. Horizontal transfer of immunity among conspecifics, i.e. both direct protection and signaling information on the immune state, potentially eliciting physiological responses, is often mediated *via* chemicals (Milutinović and Schmitt 2022). Nevertheless, little is known about the direct effects of immune stimuli like wounding and/or pathogen exposure on chemical phenotypes of individuals, which can potentially signal the immune status into the group and/or serve direct defensive functions.

Chemical cues can be crucial for healthy animals to identify diseased conspecifics across taxa (Behringer et al. 2020; Csata et al. 2017; Pull et al. 2018). Cuticular hydrocarbon (CHC) profiles may play a major role as contact cues acting as infochemicals, i.e., pheromones and allelochemicals that transfer information between individuals. CHCs cover the surface of insects and consist of complex mixtures of straight-chain, methyl-branched, and unsaturated hydrocarbons (Blomquist and Ginzel 2021). They serve as pheromones or allelochemicals for recognition of species, sex, mutualistic partners, or hosts, and for nestmate recognition and regulation of social interactions in eusocial insects (Leonhardt et al. 2016; van Zweden and d’Ettorre 2010). Many behavioral reactions to immunological and disease cues of nestmates in social insects, and even in a non-social insect species, could be directly related to changes in CHC profiles (Alciatore et al. 2021; Baracchi et al. 2012; Hernández López et al. 2017; Nielsen and Holman 2012; Richard et al. 2008). However, in an ant species, immune experiences resulted in changes in the CHCs, but their role in the observed nestmate responses were unclear, suggesting volatile chemicals or behavioral cues as mediators (Alciatore et al. 2021). These findings underline that different infochemicals can be involved in information transfer among conspecifics (Müller et al. 2020).

In addition to contact cues, volatile compounds are known to mediate interactions between organisms, both as pheromones detectable over large distances and/or with direct defensive effects against pathogens or predators (Yew and Chung 2015). For example, odors emitted by diseased brood induce hygienic behavior in bees (Swanson et al. 2009), and ant nest volatiles can have antifungal effects (Wang et al. 2015). The release of defensive secretions into the environment can be regarded as ‘external immunity’ (Cotter and Kilner 2010; Otti et al. 2014), i.e., putatively, a process of niche construction (Müller et al. 2020; Odling-Smee et al. 2013). External immunity *via* secretions has not only been found in several social species but also in various group-living (gregarious) insects, e.g. beetles from the Tenebrionidae family (Cotter and Kilner 2010). Their stink gland secretions contain mostly benzoquinones, which, along with other substances, have inhibitory effects on microbes and thus serve a hygienic function (Li et al. 2013; Yezerski et al. 2007). This phenomenon is interpreted as parental care towards larvae (Clutton-Brock 1991; Trivers 1972), but also other adults from the group could benefit from secretions of conspecifics (Gokhale et al. 2017). Acting as pheromones, benzoquinones can trigger aggregation in low concentrations, but also mediate dispersal, being repellent and autotoxic in high concentrations (Chapman 1926; Roth and Howland 1941; Sokoloff 1974). However, the cascade from internal immune experience to external immunological responses is rarely studied.

The red flour beetle, *Tribolium castaneum* (Herbst 1797) (Coleoptera: Tenebrionidae), is a non-social, but group-living insect that lives in and consumes flour, and serves as a model species (Campbell et al. 2022), also for studying resistance and immune reactions to various types of stimuli (e.g., Altincicek et al. 2008; Wegner et al. 2008). Exposure to inactivated pathogen or a sublethal dose of living pathogen has previously been shown to induce immune ‘priming’, which is a form of innate immune memory in invertebrates (Little and Kraaijeveld 2004; Netea et al. 2016; Milutinović and Kurtz 2016), leading to enhanced protection (Khan et al. 2017; Roth et al. 2009; Tate and Graham 2017), associated with strong and specific transcriptional responses in *T. castaneum* (Ferro et al. 2019; Greenwood et al. 2017). Beyond that, even after experience of wounding alone this species shows social transfer of immunity, as the interaction of naïve beetles with wounded conspecifics induces an enhanced activity of the important immune effector phenoloxidase (Peuß et al. 2015). However, it is unclear if CHCs or other infochemicals could act as cue for conspecifics to detect immune-stimulated beetles. Moreover, *T. castaneum* engages in external immune defenses, releasing benzoquinone-rich stink gland secretions into the flour shared with conspecifics (Joop et al. 2014; Prendeville and Stevens 2002; Yezerski et al. 2007). The role of the main compounds, methyl- and ethyl-1,4-benzoquinone (MBQ and EBQ, respectively) and their carrier 1-pentadecene (Loconti and Roth 1953; Villaverde et al. 2007), for inter- and intraspecific interactions in *Tribolium* beetles is well described (Blum 1981; Duehl et al. 2011; Verheggen et al. 2007; Yezerski et al. 2007). For intraspecific communication, a sex-specific function was suggested for benzoquinones in *T. castaneum*, being specifically involved in female density-dependent competition (Khan et al. 2018). However, to our knowledge, it is yet unknown how immune experiences shape overall secretion profiles, whether these responses differ between females and males, and whether potential changes are persistent over time.

We here examined changes in infochemicals of red flour beetles resulting from different immune experiences, i.e., wounding alone or in combination with the injection of heat-killed *Bacillus thuringiensis* bv. *tenebrionis* (bacterial beetle pathogen, ‘priming’ treatment) in comparison to naïve beetles. We analyzed insect extracts using gas chromatography-flame ionization detection and expected changes in (1) the CHC profiles, which may act as a cue for the immune status of conspecifics and (2) the gland secretion profiles, in particular quantities of EBQ, MBQ and 1-pentadecene, which are involved in external immunity and population regulation.

## Methods and Materials

### Model Organisms

Our study was done on the outbred strain Croatia 1 (CRO1) of *T. castaneum* which was kept at 30 ºC, 70% humidity and a 12 h:12 h light:dark cycle in plastic boxes containing heat-sterilized (75 ºC for at least 24 h) organic wheat flour (Bio Weizenmehl Type 550, dm-drogerie markt) with 5% brewer’s yeast powder (hereafter referred to as “flour”) (Milutinović et al. 2013). For this experiment, around 2,000 one-month old adult beetles were allowed to mate and oviposit for 24 h in approximately 350 g flour.

A culture of *Bacillus thuringiensis* bv. *tenebrionis* was obtained from Bacillus Genetic Stock Center (Ohio State University, USA). The species is a bacterial pathogen of beetles, and immune priming *via* injection of this bacterium has been demonstrated in previous work (e.g., Ferro et al. 2019). Heat-killed vegetative cells at 1 × 10^9^ cells mL^− 1^ used for the immune stimulation treatment were prepared as previously described (Ferro et al. 2017; Roth et al. 2009) (see Methods S1 for details).

### Immune Treatment

Ten days after oviposition, all experimental beetles were individually placed into sterile 96-well plates (Type F, Sarstedt) containing 0.08 g flour per well to minimize any injuries due to conspecific cannibalism or mating (Alabi et al. 2008; Park et al. 1965; Via 1999). One week post eclosion, virgin beetles of both sexes were divided into three treatment groups: Untreated as the handling control (‘naïve’), injected with sterile PBS (‘wounded’) and heat-killed *B. thuringiensis* bv. *tenebrionis* injected (‘primed’). For the latter two treatments, 18.4 nL (~ 20,000 cells per injection in the ‘primed’ treatment) of solution was injected into the body cavity of adults with a nanoinjector (Drummond Nanoject II) between the head and pronotum laterally towards the abdomen. Subsequently, beetles were transferred into clean 96-well plates without flour, to reduce the contamination of samples by fecal material and flour.

For the profiling of CHCs at 18 h post treatment, we prepared six samples of four beetles for each sex and treatment. The sampling time was chosen based on a previous study that reported potential transfer of the immune status within 18 h post treatment (Peuß et al. 2015). For the stink gland secretion collection at 24 and 72 h post treatment, we obtained samples of eight individual beetles per sex, treatment and time point. These two time-points were chosen to test the time course of the stink gland responses to the treatment.

### Extraction of Beetle Chemicals

For extraction of the CHCs, four beetles per sample were pooled and freeze-killed at -20 ºC. Samples were shaken in 120 µL of *n*-hexane (GC-MS grade, Merck) containing 5 µL of *n*-eicosane (99.5%, Sigma-Aldrich) solution (0.2 mg mL^− 1^ in *n*-hexane) as internal standard at 4 ºC for 10 min. After centrifugation, 100 µL of each extract was dried under reduced pressure, redissolved in 80 µL of *n*-hexane and stored at -20 ºC until analysis. The volume of *n*-hexane was scaled down accordingly in three samples, where less than four beetles were available. For extraction of stink gland secretions, individual beetles were transferred to pre-cooled tubes that were then immersed in ice water at 3 °C for 3 min to stimulate the release of secretions (Joop et al. 2014). Beetles were kept in the same tubes and were immediately freeze-killed at -20 ºC. After addition of 65 µL ice-cold acetone (LC-MS grade, Sigma-Aldrich) containing 0.05 mg mL^− 1^ *n*-octadecane (98.5%, Sigma-Aldrich) as internal standard, the tubes were shaken for 5 min at 4 °C. Following centrifugation, 50 µL of each extract were kept at -20 ºC until analysis. For each set of samples (CHCs and secretions), four blank samples without beetles were prepared using the same procedures.

### Chemical Analysis, Data Pre-Processing and Feature Identification

All samples and blanks were analyzed using a gas-chromatograph coupled with a flame ionization detector (GC-FID-2010 Plus, Shimadzu) and equipped with a VF-5ms column (30 m × 0.25 mm × 0.25 μm, with 10 m EZ-guard column, J&W Agilent Technologies). For the analysis of CHCs, 1 µL from each sample was injected with a split ratio of 2 at a constant nitrogen flow of 1.02 mL min^− 1^. The column oven temperature started at 100 °C, first increased to 230 °C at a rate of 20 °C min^− 1^, then increased to 260 °C at a rate of 5 °C min^− 1^, further increased to 277 °C at a rate of 1 °C min^− 1^, and finally increased to 320 °C at a rate of 5 °C min^− 1^, which was held for 9.9 min. For the analysis of stink gland secretions, a sample volume of 1 µL was injected with a split ratio of 3 at a constant stream of hydrogen flow of 1.13 mL min^− 1^. The column oven temperature was set at 50 ºC, held for 2 min and increased to 300 ºC at a rate of 10 °C min^− 1^, followed by a holding time of 10 min. Additionally, two alkane mix standards, C8-20 and C8-C40 (both Sigma-Aldrich), were analyzed using both chromatographic methods. Standards of MBQ (98% purity), methyl-1,4-hydroquinone (99%, both Sigma-Aldrich) and ethyl-1,4-hydroquinone (97%, Abcr GmbH, 76,187 Karlsruhe, Germany) were analyzed using the method for gland secretions. The hydroquinones are the form to which benzoquinones convert to (Yezerski et al. 2000) and could thus appear in the datasets.

Data pre-processing was done using the GCsolution Postrun software (Version 2.30.00, Shimadzu), and retention time alignment of peaks in R (version 4.0.3, R Core Team 2020) using the package *GCalignR* (Ottensmann et al. 2018); for feature selection criteria and details on settings see Methods S2 in the Supplementary Material. Data were normalized to the internal standard and relative feature values calculated (proportion of total amount per sample). For identification, the retention times of peaks from the alkane mix standards were used to calculate the retention index (RI) of peaks according to Kováts (1958). Putative identification of CHCs was done by comparing the RIs with those published in previous studies (Alnajim et al. 2019; Awater-Salendo et al.; 2020; Lockey 1978). Benzoquinones as well as other major secretion compounds previously described in *T. castaneum* glandular secretions were identified by comparing their RIs to previous studies (Lehmann 2015; Li et al. 2013) and by comparisons to the analyzed commercial standards.

### Statistical Analyses

All statistical analyses were done using R (version 4.0.3, R Core Team 2020). To visualize dissimilarities between groups of beetles, non-metric multidimensional scaling (NMDS) based on pairwise Kulczynski distances (package *vegan*, version 2.5-7, Oksanen et al. 2020), was applied to the relative datasets (Wisconsin-square-root-double-standardized) of CHCs and secretions, respectively. After testing assumptions, permutational multivariate analysis of variance (PERMANOVA) was performed to test for the influence of treatment on the dissimilarities, partly combined with post hoc comparisons (Martinez Arbizu 2020) including corrections (Benjamini and Hochberg 1995) (see Methods S3). Analyses of treatment effects were done separately for sexes and in secretion datasets also separately for time points. Additionally, sex differences in the CHC profiles of naïve females *versus* naïve males were addressed using PERMANOVA.

Responses of CHC profiles of females and males to treatments were visualized in heatmaps using the package *pheatmap* (Kolde 2019). Thus, log_2_ fold change values were calculated for all CHCs and replicates in comparison to the median value of the naïve female or male beetles and data plotted as heatmaps. Moreover, heatmaps comparing CHC profiles of naïve females and naïve males were generated based on z-scores for each CHC. All heatmap plots included Euclidean cluster analysis.

For the secretions, we additionally identified features most important for profile differences between treatment groups within females and males 24 h after treatment using a conditional random forest classification on the proportional data (for details see Methods S3) (De Moraes et al. 2014; Strobl et al. 2009a, b). The relative amounts of these features, as well as the absolute amounts of MBQ, EBQ and their carrier 1-pentadecene (1-C15-ene), were analyzed for an influence of treatment within females and males at both time points. Absolute amounts were chosen for these three compounds as their biological activity is known to be concentration dependent (Joop et al. 2014; Verheggen et al. 2007). We tested the model assumptions (see Methods S3), and either fitted linear models with appropriate transformations revealed using the package *bestNormalize* (Peterson 2021), or performed non-parametric *Kruskal-Wallis tests*. When significant treatment effects were founds, *Benjamini-Hochberg-corrected post hoc* comparisons were performed between treatment groups.

## Results

### Cuticular Hydrocarbons

In total, 20 CHCs were provisionally identified in the dataset. The suggested identities comprised seven *n*-alkanes (C25-C31), six 3- and 4-methyl-branched alkanes and seven internally methyl-branched alkanes, including one dimethyl alkane (see Supplementary Information Table S1 for details).

**Fig. 1:**
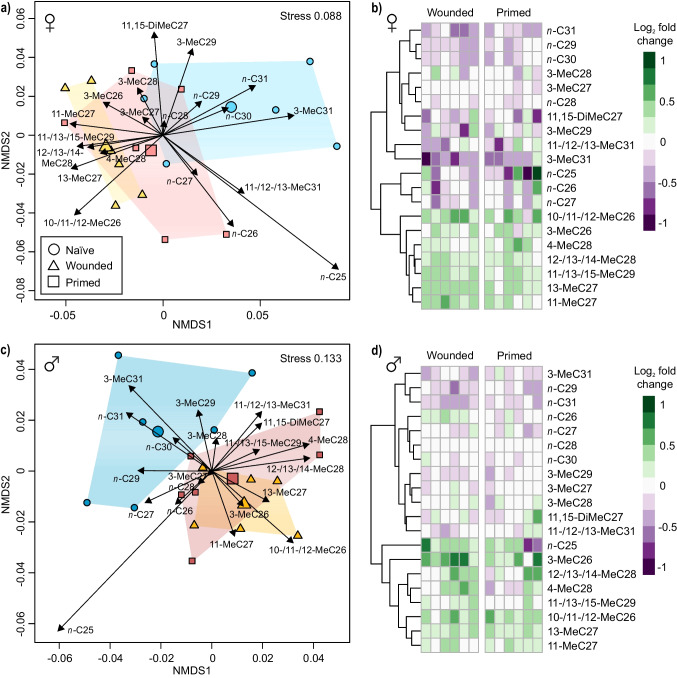
NMDS plots (**a**, **c**) and heatmaps (**b**, **d**) of cuticular hydrocarbon (CHC) profiles from female (**a**, **b**) and male (**c**, **d**) beetles of *Tribolium castaneum*. Beetles (*N* = 6 per sex and treatment) were untreated (‘Naïve’), or treated by injection of phosphate buffered saline (PBS; ‘Wounded’) or of PBS with heat-killed *Bacillus thuringiensis* bv. *tenebrionis* (‘Primed’) 18 h before extraction. All plots are based on relative amounts of 20 individual CHCs. For NMDS plots, datasets were transformed with Wisconsin square root double standardization and dissimilarities are based on pairwise Kulczynki distances. Data points represent profiles of individual beetles, larger symbols show centroids for treatment groups. Arrows range from zero to positions of expanded weighted average scores of CHCs. Heatmaps show increase and decrease in the proportion of individual CHCs in wounded and primed beetles in relation to the median value of naïve beetles as log_2_ fold change. Clustering of CHCs is based on Euclidean distances

Significant immune treatment effects on the CHC profile were found for both sexes (Table 1). In both females and males, the CHC profiles of naïve beetles differed from those of immune-treated beetles, but separated more strongly from profiles of wounded than of primed beetles in NMDS plots (Fig. 1a, c). Accordingly, in *post hoc* tests on datasets of both females and males, CHC profiles of naïve beetles differed significantly from those of wounded, but not from those of primed beetles, while CHC profiles of wounded and primed beetles were similar (Table 1). As inferred from the CHC scores in the NMDS plot and heatmaps showing treatment-related differences relative to naïve beetles, higher proportions of C26-C29 methyl alkanes characterized CHC profiles of wounded and primed beetles in comparison to those of naïve beetles in both females (Fig. 1a, b) and males (Fig. 1c, d). The CHC profiles of naïve beetles had higher proportions of C29-C31 *n*-alkanes and also of 3-MeC31 (Fig. 1).

**Table 1:** Effects of treatment on the composition of CHC profiles in female and male *Tribolium castaneum* beetles in a permutational analysis of variance (overall tests and pairwise comparisons)

	*F*	*R*^*2*^	*P*
Treatment (females)	2.5	0.25	**0.023**
naïve vs. wounded	4.8	0.33	**0.006**
naïve vs. primed	2.0	0.17	0.153
wounded vs. primed	0.6	0.06	0.630
Treatment (males)	2.4	0.24	**0.009**
naïve vs. wounded	3.8	0.28	**0.009**
naïve vs. primed	2.1	0.18	0.110
wounded vs. primed	1.2	0.10	0.317

Beetles (*N* = 6 per sex and treatment) were untreated (‘naïve’), or treated by injection of phosphate buffered saline (PBS; ‘wounded’) or of PBS with heat-killed *Bacillus thuringiensis* bv. *tenebrionis* (‘primed’) 18 h before extraction. The datasets included relative values of 20 CHCs and were transformed with Wisconsin square root double standardization. Dissimilarities are based on pairwise Kulczynki distances. Significant *P*-values shown in bold

NMDS plots of CHC profiles combining females and males resulted in a clear separation of the sexes, visible across treatments (Supplementary Information Fig. S1a). When only naïve females *versus* males were compared without potentially interfering treatment effects, this was mirrored in a significant difference (PERMANOVA; *F* = 13.5, *R²* = 0.58, *P* = 0.002). Overall, the same CHCs as for separation of naïve from immune treated beetles (Fig. 1) were involved in separation of females and males (Fig. S1). Most obviously from NMDS plot and heatmap, the CHC profiles of female beetles were characterized by higher proportions of C26-C28 methyl alkanes, especially of the cluster comprising 10-/11-/12-MeC26, 3-MeC27 and 13-MeC27 (Fig. S1b, c). In contrast, male CHC profiles contained higher proportions of C30 and C31 *n*- and methyl alkanes, especially of the cluster comprising *n*-C30 and *n*-C31, together with 3-MeC29 (Fig. S1b, c).

### Stink Gland Secretions

For analyses of the dataset acquired 24 h after treatment, 26 chemical features were included, of which 24 were also considered in the dataset acquired 72 h after treatment (Supplementary Information Table S2). The benzoquinones MBQ and EBQ could clearly be identified, but the corresponding hydroquinones were not detected. The other five identified features were alkenes with one or two double bonds (see Table S2 for details of identification of individual features). The benzoquinones together with 1-C15-ene made up the major part of the overall secretion composition (Table S2).

**Fig. 2:**
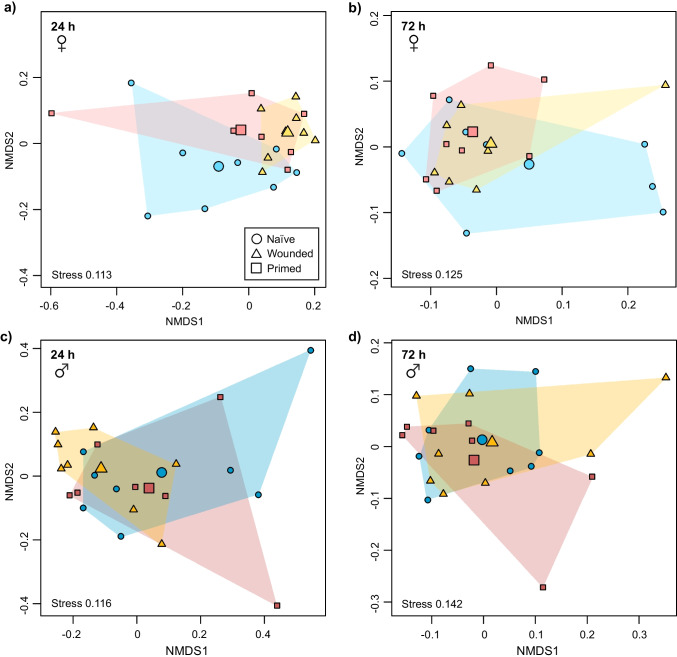
NMDS plots of glandular secretion profiles from female (**a**, **b**) and male (**c**, **d**) beetles of *Tribolium castaneum*, 24 h (**a**, **c**) and 72 h (**b**, **d**) after immune treatments. Beetles (*N* = 7–8 per sex and treatment) were untreated (‘Naïve’), or treated by injection of phosphate buffered saline (PBS; ‘Wounded’) or of PBS with heat-killed *Bacillus thuringiensis* bv. *tenebrionis* (‘Primed’). Plots are based on relative amounts of (**a**, **c**) 26 or (**b**, **d**) 24 individual chemical features released from glands. Datasets were transformed with Wisconsin square root double standardization. Dissimilarities are based on pairwise Kulczynki distances. Data points represent profiles of individual beetles, larger symbols show centroids for treatment groups

For secretion profiles of female beetles 24 h after treatment, the treatment effect was marginally non-significant (PERMANOVA, *F* = 2.0, *R²* = 0.17, *P =* 0.057). As visible in the NMDS plot, this tendency resulted mainly from separation of naïve and wounded females (Fig. 2a, Supplementary Information Fig. S2). Analyzing male beetles sampled 24 h after treatment, the composition of the overall secretions seemed visually quite similar in all three treatment groups (Fig. 2c, Supplementary Information Fig. S3), and there was no significant treatment effect (*F* = 1.4, *R²* = 0.12, *P =* 0.180). To explore if individual features from the profiles respond to immune challenge in females or males, random forest analysis was applied for both sexes separately. Random forest analysis of female beetles revealed one unidentified feature, RI 1015, as important for separation of treatment groups (Supplementary Information Fig. S4a), although it comprised only < 0.1% of the total glandular secretions (Table S2). The relative amount of this feature was significantly influenced by treatment in females (*Kruskal-Wallis test*; 2 df, *χ*^*2*^ = 9.84, *P* = 0.034; males: 2 df, *χ*^*2*^ = 1.72, *P* = 0.490), being lower in both primed and wounded beetles compared to naïve ones (Fig. S4b). The unidentified feature RI 1473 was suggested as important in males (Supplementary Information Fig. S5a), appearing in only few male beetle samples (Fig. S5b) and overall only < 0.1% of the total secretions (Table S2). The relative amount of this feature was significantly influenced by treatment in males (*Kruskal-Wallis test*; 2 df, *χ*^*2*^ = 9.27, *P* = 0.034; females: 2 df, *χ*^*2*^ = 3.43, *P* = 0.370), being higher in wounded than in naïve beetles (Fig. S5b). In the dataset acquired 72 h after treatment, neither females (PERMANOVA, *F* = 0.6, *R²* = 0.105, *P =* 0.802) nor males (*F* = 0.6, *R²* = 0.06, *P =* 0.854) revealed statistical or visual effects of treatments on the secretion profiles (Fig. 2b, d) and thus further random forest analysis was not performed. Visually comparing female and male profiles across all treatments in NMDS plots, potential effects seemed marginal and inconsistent at both time points (Supplementary Information Fig. S6).

**Fig. 3:**
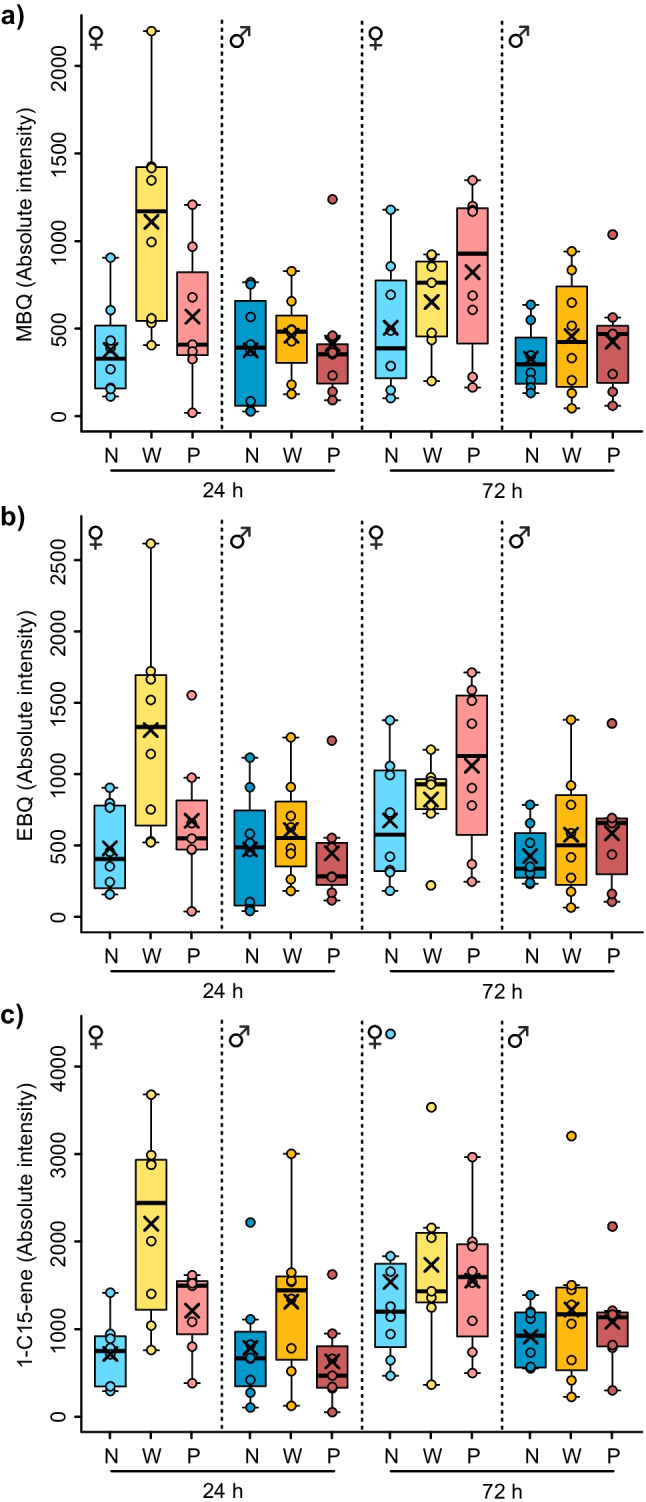
Amount of (**a**) methyl-1,4-benzoquinone (MBQ), (**b**) ethyl-1,4-benzoquinone (EBQ) and (**c**) 1-pentadecene (1-C15-ene) from glandular secretions of female and male beetles of *Tribolium castaneum* at two time points (24 h, 72 h) after immune treatments. Beetles (*N* = 7–8 per sex, treatment and time point) were untreated (naïve, ‘N’), or treated by injection of phosphate buffered saline (PBS; wounded, ‘W’) or of PBS with heat-killed *Bacillus thuringiensis* bv. *tenebrionis* (primed, ‘P’). Boxes show 25th and 75th percentiles, lines are medians, crosses are means, whiskers mark minimum and maximum within 1.5-fold interquartile ranges. Dots represent values of individual beetles

Furthermore, we compared the absolute amounts of the major compounds MBQ, EBQ and their carrier 1-C15-ene. In the secretions acquired after 24 h, an effect of treatment was only significant for females, but not for males in these three compounds (Table 2; Fig. 3). In *post hoc* tests, the absolute amounts of all three compounds differed significantly between naïve and wounded females (Table 2), being higher in wounded individuals (Fig. 3). The amounts of MBQ and EBQ tended to be higher in wounded than in primed females and this difference was significant for 1-C15-ene (Table 2; Fig. 3). In the dataset acquired 72 h after treatment, no treatment effects were found in females or males (Table 2). Interestingly, in secretions collected after 72 h the benzoquinones in primed, but not in wounded females, reached the highest average amounts (Fig. 3a, b).

**Table 2:** Effects of treatment on absolute amounts of methyl-1,4-benzoquinone (MBQ), ethyl-1,4-benzoquinone (EBQ), and 1-pentadecene (1-C15-ene) from glandular secretions of female and male beetles of *Tribolium castaneum* in linear models (overall tests) and *Tukey HSD* post hoc tests (pairwise comparisons)

	MBQ			EBQ			1-C15-ene		
24 h after treatment	df	*F/t*	*P*	df	*F/t*	*P*	df	*F/t*	*P*
Treatment (females)	2	5.36	**0.014**	2	5.12	**0.016**	2	9.02	**0.002**
naïve vs. wounded	1	3.16	**0.015**	1	3.07	**0.018**	1	4.23	**0.001**
naïve vs. primed	1	0.79	0.439	1	0.70	0.492	1	1.69	0.107
wounded vs. primed	1	2.27	***0.052***	1	2.27	***0.052***	1	2.40	**0.040**
Treatment (males)	2	0.37	0.694	2	0.60	0.559	2	1.31	0.292
72 h after treatment									
treatment (females)	2	1.13	0.343	2	1.23	0.313	2	0.12	0.887
treatment (males)	2	0.03	0.972	2	0.05	0.956	2	0.09	0.912

Beetles (*N* = 7–8 per sex, treatment and time point) were untreated (‘naïve’), or treated by injection of phosphate buffered saline (PBS; ‘wounded’) or of PBS with heat-killed *Bacillus thuringiensis* bv. *tenebrionis* (‘primed’) 24 or 72 h before extraction. Significant *P*-values shown in bold, marginally significant *P* values in bold and italics. Column *F*/*t* shows *F* value in linear models and *t* value in *Tukey HSD* post hoc tests

## Discussion

As group-living animals that share the same food resources and live in proximity, flour beetles are prone to increased disease transmission. We investigated the influence of different immune stimuli on profiles of different infochemicals in *T. castaneum.* Wounding led to similar changes in CHC patterns of females and males, potentially involved in transferring information on the immune status within the group. Wounding-induced changes in gland secretion compounds that could directly mediate external immunity and behavioral responses were mainly restricted to females. The response to priming by injecting with heat-killed bacteria was lower than to wounding across both types of infochemicals, emphasizing the complexity of physiological responses, especially regarding internal (individual) *versus* external (group) immunity.

Comparing different types of immune stimulation, in a previous study wounding alone (i.e., pricking) resulted in weaker immune gene expression than treatment with live *B. thuringiensis* bv. *tenebrionis* (Behrens et al. 2014). In our work, surprisingly, the response of *T. castaneum* to priming was weaker than the response to wounding across both types of infochemicals, CHCs and quinones. Firstly, wounding may quickly establish a transient physiological state of heightened ‘alertness’, leading to responses with quinones as broadly-acting antimicrobials (Joop et al. 2014; Pedrini et al. 2015; Sawada et al. 2020), and potentially being displayed to conspecifics in changed CHC profiles. Meanwhile, immune priming *via* injecting heat-killed bacteria may carry more information and induce additional physiological and immunological responses that mediate more tailored defenses against certain types of pathogens (Ferro et al. 2019; Roth et al. 2009). Secondly, potential competition for tyrosine precursors between both the quinone production and the phenoloxidase cascade activated upon bacterial challenge (Behrens et al. 2014; Joop et al. 2014; Otti et al. 2014), may result in a trade-off between external and internal immunity. While external immunity by quinones also confers protection to adjacent conspecifics (Cotter and Kilner 2010; Gokhale et al. 2017), initially prioritizing individual internal immunity may be beneficial for this non-social species. The aforementioned possible reasons, together with the finding that 72 h after treatment primed beetles had the highest average amounts of quinones, hint to a delay or shift in immunological processes rather than absence of responses in primed beetles. This may indicate a more durable enhanced protection representing a ‘primed’ physiological state. Further analyses are needed to explore immune responses in CHCs and secretion compounds and their associated metabolic processes upon wounding *versus* priming over time.

The CHC profiles of the beetles were hypothesized to be altered upon immune stimulation *via* wounding and/or priming. Indeed, we found significant differences in the CHC profiles of naïve *versus* wounded females and males. These changed profiles may act as a potential cue for the detection of immune experience in conspecifics, leading to the previously described social transfer of immunity (enhanced phenoloxidase activity) from wounded to naïve *T. castaneum* during cohabitation (Peuß et al. 2015). Moreover, in that study, cohabitation with wounded beetles resulted in reduction in expression of two heat shock protein genes, *Hsp8*3 and *Hsp90*, in naïve conspecifics (Peuß et al. 2015) within the same time span, in which profile changes occurred in the present study. As the molecular chaperone HSP90 has been hypothesized to serve as an evolutionary capacitor by mediating the storage and release of cryptic genetic variation (Rutherford and Lindquist 1998), such down-regulation could have the potential to lead to increased adaptability. However, a causal relationship between changes in CHCs and transfer of information on wounding, including the consequences for social immunity and evolution, needs to be proven in additional bioassays.

The CHC profiles of immune-stimulated beetles in the present study, especially the wounded ones, were characterized by higher proportions of methyl alkanes. Also in studies on other insect species, specifically methyl alkane CHCs were altered by immune challenge (Alciatore et al. 2021; Baracchi et al. 2012; Richard et al. 2008), and could therefore contribute to social transfer of immune status information. Despite higher metabolic costs for these CHCs (Nelson 1993), a generally more important role of methyl alkanes in comparison to linear *n*-alkanes as intraspecific recognition cues is well documented in social insects (van Zweden and d’Ettorre 2010), and was found for interspecific interactions of *T. castaneum* larvae (Awater-Salendo et al. 2020). Furthermore, in larvae of *T. castaneum*, wounding caused an upregulation of multiple candidate fatty acid synthase genes (Supplementary Information Table S3; Behrens et al. 2014), which are involved in the synthesis of methyl alkanes (Blomquist and Ginzel 2021; Holze et al. 2021). Although immune responses in *T. castaneum* can vary between larvae and adults (Tate et al. 2021), the wounding-induced alterations in methyl alkanes from CHC profiles of adults observed here could potentially result from an active process of similar upregulation of genes involved in their synthesis as found for the larvae. Moreover, changes in fatty acid synthesis might alter eicosanoids, C20 unsaturated fatty acid metabolites which are crucial in mediating cellular immune responses in insects (Kim and Stanley 2021), suggesting that also internal immune signaling could be influenced by treatments. Taken together, methyl-branched CHCs are likely an important factor for intraspecific transfer of immune information in both larvae and adults of *T. castaneum*, and the genetic basis should be further explored in detail.

Furthermore, we could demonstrate a sexual dimorphism in the CHC profiles of naïve *T. castaneum* adults, which is also well documented in various other invertebrate species (Jallon and David 1987; Müller and Müller 2016; Thomas and Simmons 2008). However, CHC profiles of females and males changed in similar directions upon immune stimulation in *T. castaneum*. In contrast, in the non-social species *T. molitor* changes of the CHC profiles after immune challenge were sex-specific, and the resulting (sexual) signals towards conspecifics resulted in advantages for the wounded males, interpreted as terminal investment (Nielsen and Holman 2012). The similarity in the CHC profile changes across sexes in the present study, may be beneficial for an effective transfer of information into the group, and could thus be regarded as ‘social trait’. To investigate the species-specific functions of changes in CHC profiles, comparative studies on females and males of various eusocial *versus* non-social insect species are needed.

Flour beetles perform niche construction by secreting quinones-rich stink gland secretions into the flour, which influences microbial growth (Sokoloff 1974; Van Wyk et al. 1959) as a form of external immunity (Joop et al. 2014; Otti et al. 2014). Considering this as parental care (Trivers 1972; Clutton-Brock 1991), we expected that adult females and/or males may modulate their secretions upon detection of pathogen risks to protect their offspring which lack the glands. We found an increase in the absolute amount of the benzoquinones (MBQ, EBQ) and their carrier 1-C15-ene in the secretions of wounded females 24 h after the treatment. The finding that particularly females may engage in niche construction fits theory, proposing more resource investment in immunity and longevity (Bateman 1948; Rolff 2002) or into brood protection (Trivers 1972) in females than in males. However, these effects lasted for less than 72 h after immune treatments. Potential metabolic costs associated with quinone production and individual survival costs caused by autotoxicity of quinones in high concentrations (Gokhale et al. 2017; Joop et al. 2014; Yezerski et al. 2004), may explain the transience of this effect. Thus, there is likely a complex trade-off between benefits and various individual cost of quinone production, determining the time course of responses to immune experience. This study underlines the importance to distinguish between sexes and time points when exploring responses to immune challenge, potentially influencing niche construction.

Apart from serving as antimicrobials, benzoquinones and alkenes in stink gland secretions also lead to dose-dependent behavioral responses in conspecifics of *Tribolium* beetles (Suzuki 1985; Verheggen et al. 2007). For example, together with an aggregation pheromone, 4,8-dimethyldecanal, benzoquinones and alkenes mediate dispersal (Duehl et al. 2011; Ogden 1969; Verheggen et al. 2007). If wounding occurs at high population densities, an enhanced secretion of benzoquinones can signal overcrowding (Faustini and Burkholder 1987; Verheggen et al. 2007). Females appear particularly sensitive to perceive certain chemical cues, for example the secretions of other females in the flour, responding by upregulation of their own quinone production (Khan et al. 2018). High amounts of quinones can even suppress the fecundity of conspecifics, which has consequences for population density regulation (Khan et al. 2018; Sonleitner and Gutherie 1991). Thus, changes in absolute quinone amounts after immune stimulation in females may act as sex-specific signals towards conspecifics, as described for pheromones in *T. molitor* (Nielsen and Holman 2012; Rantala et al. 2002; Sadd et al. 2006). The overall gland secretion profile composition did not show apparent changes in response to immune stimulation. Only two unidentified features at very low relative concentration, determined by random forest analysis, revealed sex-specific treatment effects. Identification of these features may help characterize any biological relevance of their modulation. Further, quantification of amounts of quinones and other features actually present in the flour environment could contribute to elucidate how the beetles regulate their environment and/or population density *via* their stink gland secretions.

In conclusion, our study highlights the potential role of chemical profiles in mediating the intertwined processes of immunity and niche construction in the red flour beetle. Immune stimulation triggered changes in potentially important infochemicals which can have fitness consequences for both the individual and the group. Furthermore, our results emphasize the high specificity of responses towards different types of immune stimuli and the importance to focus on sex-specific functions of chemicals. More generally, regarding the special lifestyle of a group-living but non-social insect, these results bear fundamental relevance for questions of common and specific mechanisms of disease control in groups and societies across taxa.

## Supplementary Information

Below is the link to the electronic supplementary material.

ESM 1 (XLSX 92.6 KB)

ESM 2 (R 26.1 KB)

ESM 3 (PDF 3.39 MB)

## Data Availability

Raw data and R codes are provided in the Supplementary Information. The GC-FID raw data will be released upon publication of the paper in the MetaboLights repository (Haug et al. 2013, 2020), being available with the accession number MTBLS2277 at www.ebi.ac.uk/metabolights/MTBLS2277.
